# First record of translocation in Culicidae (Diptera) mitogenomes: evidence from the tribe Sabethini

**DOI:** 10.1186/s12864-019-6069-3

**Published:** 2019-09-27

**Authors:** Camila Lorenz, João Marcelo Pereira Alves, Peter Gordon Foster, Maria Anice Mureb Sallum, Lincoln Suesdek

**Affiliations:** 1 Department of Epidemiology, School of Public Health, University of Sao Paulo, Av. Dr. Arnaldo, 715, São Paulo, CEP 05509-300 Brazil; 2 Department of Parasitology, Institute of Biological Science, University of Sao Paulo, Av. Prof. Lineu Prestes, 1374, São Paulo, SP 05508-000 Brazil; 3 Department of Life Sciences, Natural History Museum, Cromwell Rd, London, UK; 4 Butantan Institute, Av. Vital Brazil 1500, Butanta, São Paulo, SP CEP 05503-900 Brazil; 5 Institute of Tropical Medicine, University of Sao Paulo, Av. Dr. Enéas de Carvalho Aguiar 470, Jardim América, São Paulo, SP CEP 05403-000 Brazil

**Keywords:** Mitochondria, Insect, Rearrangement, Autapomorphy, Neotropical

## Abstract

**Background:**

The tribe Sabethini (Diptera: Culicidae) contains important vectors of the yellow fever virus and presents remarkable morphological and ecological diversity unequalled in other mosquito groups. However, there is limited information about mitochondrial genomes (mitogenomes) from these species. As mitochondrial genetics has been fundamental for posing evolutionary hypotheses and identifying taxonomical markers, in this study we sequenced the first sabethine mitogenomes: *Sabethes undosus, Trichoprosopon pallidiventer, Runchomyia reversa, Limatus flavisetosus*, and *Wyeomyia confusa*. In addition, we performed phylogenetic analyses of Sabethini within Culicidae and compared its mitogenomic architecture to that of other insects.

**Results:**

Similar to other insects, the Sabethini mitogenome contains 13 protein-coding genes, 22 transfer RNA genes, two ribosomal RNA genes, and a control region. However, the gene order is not the same as that in other mosquitoes; the tyrosine (Y) and cysteine (C) tRNA genes have translocated. In general, mitogenome rearrangements within insects are uncommon events; the translocation reported here is unparalleled among Culicidae and can be considered an autapomorphy for the Neotropical sabethines.

**Conclusions:**

Our study provides clear evidence of gene rearrangements in the mitogenomes of these Neotropical genera in the tribe Sabethini. Gene order can be informative at the taxonomic level of tribe. The translocations found, along with the mitogenomic sequence data and other recently published findings, reinforce the status of Sabethini as a well-supported monophyletic taxon. Furthermore, *T. pallidiventer* was recovered as sister to *R. reversa*, and both were placed as sisters of other Sabethini genera (*Sabethes*, *Wyeomyia*, and *Limatus*).

## Background

Insect mitochondrial DNA (mtDNA or the mitogenome) consists of a compact circular molecule usually 15–18 kb in length. It encodes 37 genes: 13 protein-coding genes (PCGs), two ribosomal RNA (rRNA) genes, and 22 transfer RNA (tRNA) genes. All of these genes are involved in translation of the PCGs [1]. This complement of 37 genes is highly conserved in metazoan animals, with rare exceptions [2]. The relative positions of PCGs and rRNA genes show limited variation among different phyla compared with tRNA genes [3]. For this reason, it has been proposed that tRNA gene rearrangements may be useful phylogenetic markers [1, 4]. The ancestral insect mitogenome differs from the ancestral arthropod mitogenome only by the position of one tRNA gene [5]. Although notable deviations from the ancestral insect mitogenomes in structure, gene arrangement, and gene content have been reported within insects, it is clear by analysing the numerous mitogenomes currently available that these exceptions are found only in highly derived portions of the insect phylogeny [1].

Among insects, one of the most studied groups is the family Culicidae (mosquitoes) because of their medical and veterinary importance worldwide [6]. A total of 3564 species of Culicidae are currently recognised [7], classified in two subfamilies and 113 genera. There are approximately 1 hundred mitogenomes available representing several mosquito species, mainly from medically important genera of pathogen-vectoring species such as *Aedes*, *Anopheles* and *Culex* [8]. All currently available Culicidae mitogenomes have similar gene order and arrangement to the ancestral insect mitogenome [8–11], with the exception of an inversion reported by Beard et al. [12]. Despite their importance as transmitters of the yellow fever virus, to the best of our knowledge, there is only one study addressing the phylogenetic relationships within the tribe Sabethini, and the relationship between sabethines and other culicids using mitogenomes [13]. Although Aragão et al. [13] did not address gene arrangements within the mitogenome, there are some unique characteristics of the mitochondria of sabethines that separate them from other culicids. Members of this tribe are among the most morphologically and biologically diverse of all mosquitoes, and present greatest species diversity in the tropics [14]. In general, immature Sabethini species use as their habitat phytotelma, which can be formed by leaves, flowers, bamboo internodes, bromeliads, pitcher plants, and many other plant structures [14, 15].

Although long recognized as a monophyletic group, the taxonomic placement of sabethines within the Culicidae has varied, as well as the genus relationships within the tribe (see Judd [14]). Some advances have been made in the classification of this tribe [16–20], but the Sabethini as a whole is far from being organized into natural groups [14]. Results of the analyses corroborate the classification proposed by Judd in recognizing both the tribe Sabethini and Neotropical sabethines as monophyletic groups [21]. Currently, there are two major groups within the Sabethini, “New World” (Neotropical) and “Old World” (mainly Oriental/Australian) groups [14, 22] with approximately 700 valid species assigned into 13 genera [7]. Regarding the Sabethini taxonomic placement within Culicidae, there is considerable controversy in the literature. Harbach and Kitching [21] placed tribe Sabethini close to tribe Culicini, based on morphological characters. In contrast, Reidenbach et al. [23], using morphological and genetic data, recovered the Sabethini and Aedini tribes as closely related.

In light of these knowledge gaps and this controversial situation, we started investigating the phylogeny of Culicidae at the genus level based on mitogenomes (Lorenz et al. MS submitted). Results corroborated the monophyly of Sabethini and incidentally unveiled tRNA translocations in some samples of that tribe. Then, we decided to perform a new study, now at the species level, to further understand the information obtained from sabethine mitochondrial data. The goals of the current study were to: 1) verify the arrangement and order of genes in the mitogenomes of Sabethini (*Sabethes undosus, Trichoprosopon pallidiventer, Runchomyia reversa, Limatus flavisetosus*, and *Wyeomyia confusa*) and 2) establish phylogenetic relationships among Sabethini species and with other culicids. Our results showed that the gene order contains phylogenetic information that complements the monophyly of the tribe Sabethini.

## Results

**Fig. 1:**
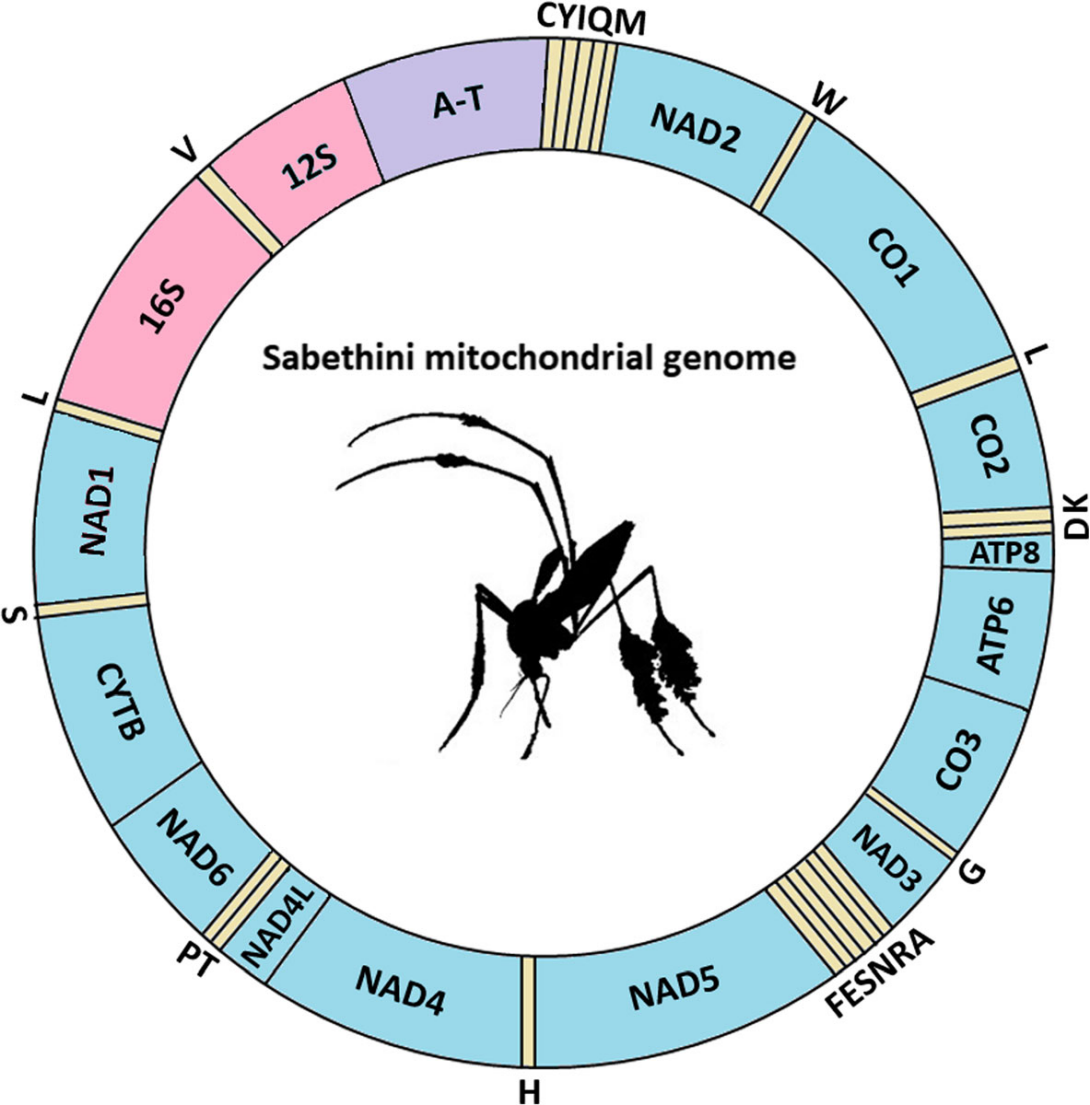
Graphical representation of the arrangement and gene order of Sabethini mitochondrial genomes. The tRNA genes are indicated by letters according to the IUPAC-IUB abbreviations for amino acids. The genes in blue represent the PCGs; in pink are the ribosomal genes (small and large subunits); and in purple, the AT-rich control region

General characteristics of the mitogenome of the five newly sequenced Sabethini species from this study are in Table 1. The mitochondrial genomes are similar to those of three *Sabethes* species studied by Aragão et al. [13], ranging from 15,302 to 16,037 bp in length. Length variation is mainly due to differences in the length of control regions in Sabethini species. In addition to this AT-rich control region, the mitogenomes also contained 37 genes: 13 PCGs, 22 tRNA genes, and two rRNA genes (Fig. 1).

**Table 1:** General characteristics of Sabethini mitogenomes obtained in this study

Species	Length (bp)	1st codon AT%^a^	2nd codon AT%^a^	3rd codon AT%^a^
*Sabethes undosus*	15,334	72.3	67.7	95.4
*Limatus flavisetosus*	15,663	72.0	68.4	96.5
*Wyeomyia confusa*	15,456	71.5	67.7	94.1
*Trichoprosopon pallidiventer*	16,037	71.0	67.4	94.8
*Runchomyia reversa*	15,302	71.7	67.6	92.8

^a^AT% per codon position were calculated only for the 13 protein-coding genes

**Fig. 2:**
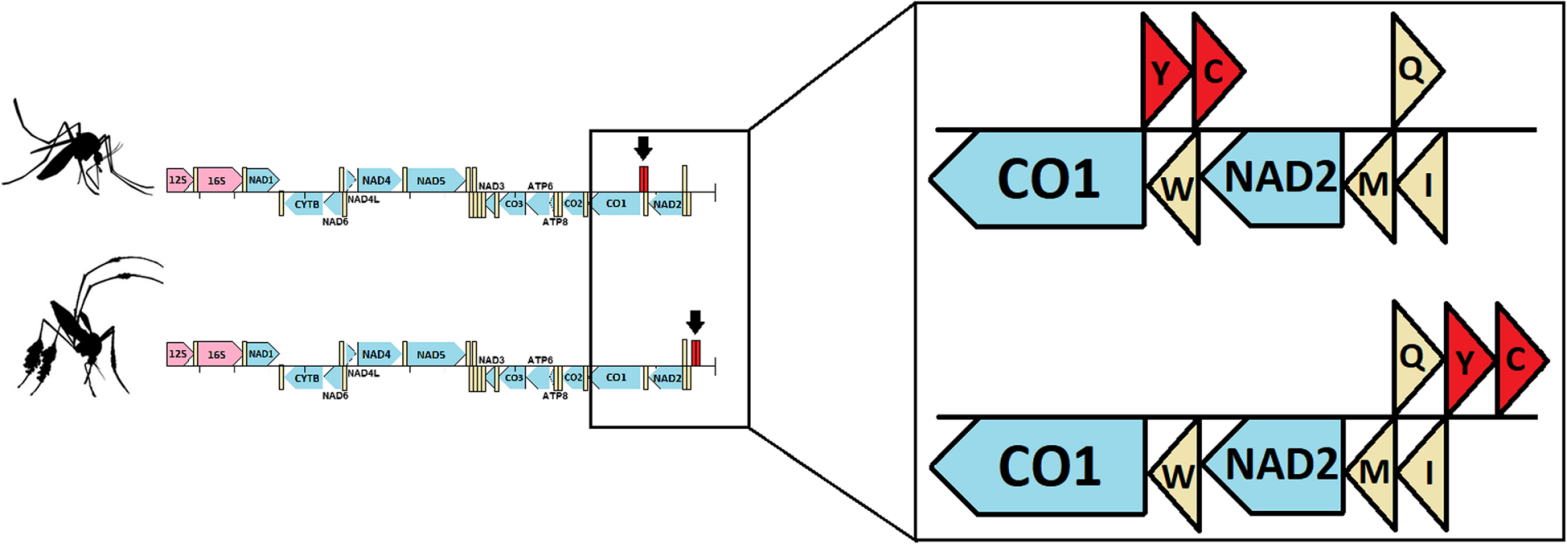
Schematic diagram showing the position and orientation of mitochondrial genes: Above - most of the Culicidae mtDNA; Below - representatives of the tribe Sabethini (*Sabethes, Limatus, Wyeomyia, Runchomyia*, and *Trichoprosopon*). Arrows indicate the location of C and Y tRNA genes. The AT-rich control region was omitted from the representation

Gene order in the mitogenomes in the Sabethini differed from other mosquito species. Tyrosine (Y) and cysteine (C) tRNAs are translocated (Fig. 2). In the available mitogenomes from other groups, Y and C are located between *COI* and tRNA-W genes, whereas in the Sabethini, they are between the tRNA-I gene and the AT control region. Our results suggest that this translocation is an autapomorphy of the Neotropical sabethines.

**Fig. 3:**
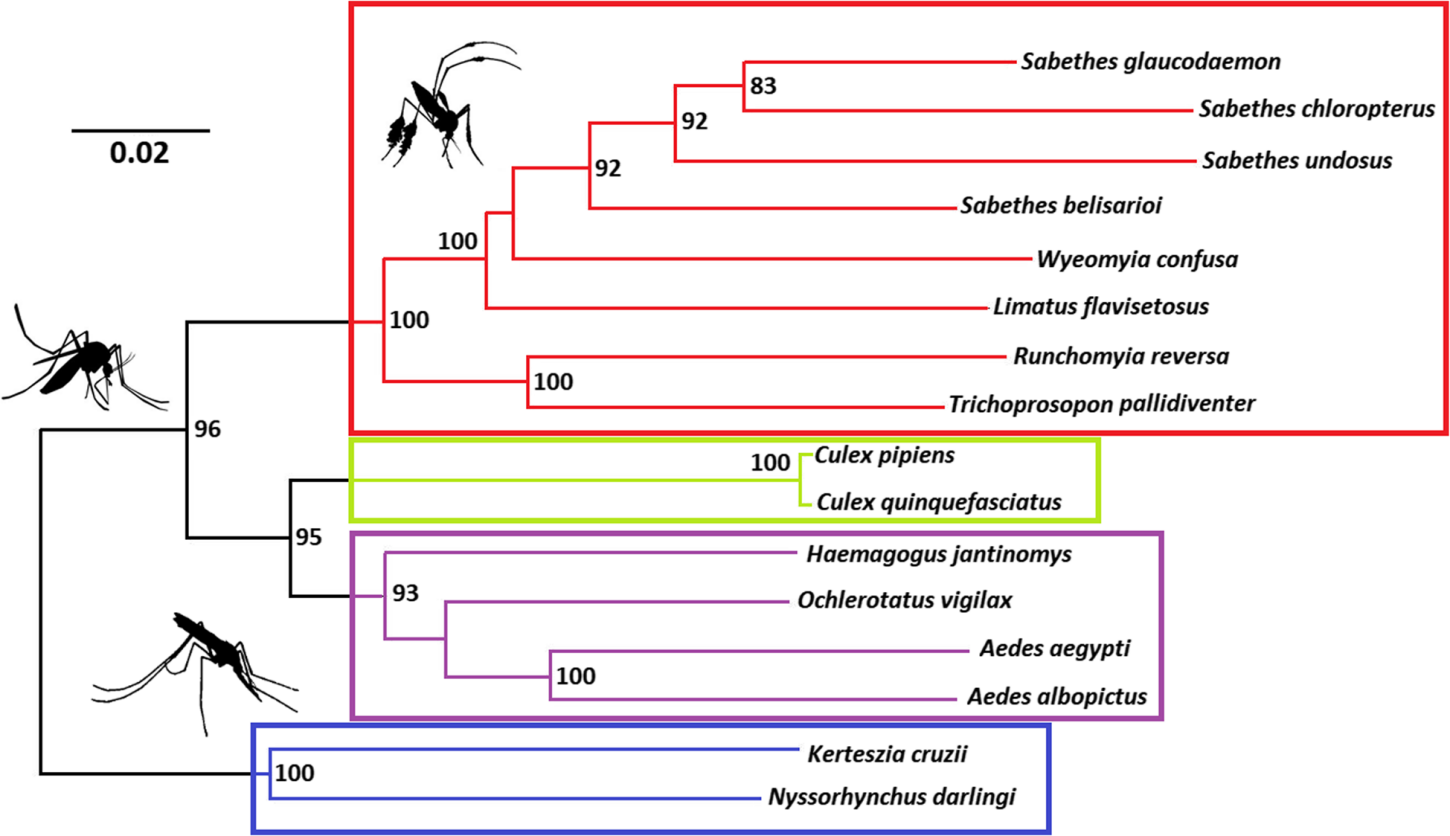
Phylogenetic tree recovered using the ML and BI inference methods employing amino acids of the 13 PCGs in the mitogenomes of 16 Culicidae species. Highlighted boxes enclose (from top to bottom) Sabethini, Culicini, Aedini, and Anophelinae. Bootstrap support values (left) and Bayesian posterior probabilities (right, %) higher than 80 are shown on the branches of the tree

Results of phylogenetic analyses showed that the representative species of Sabethini are monophyletic (Fig. 3). The phylogenetic analysis based on BI and ML recovered identical tree topologies, with variations in the statistical support of some branches. The three *Sabethes* species available in GenBank clustered with *S. undosus*. The strongly supported clade composed of *T. pallidiventer* and *R. reversa* was sister to the remaining sabethines. All clades corroborate the taxonomic classification of Sabethini proposed by Reidenbach et al. [23] based on morphology and sequence data.

**Fig. 4:**
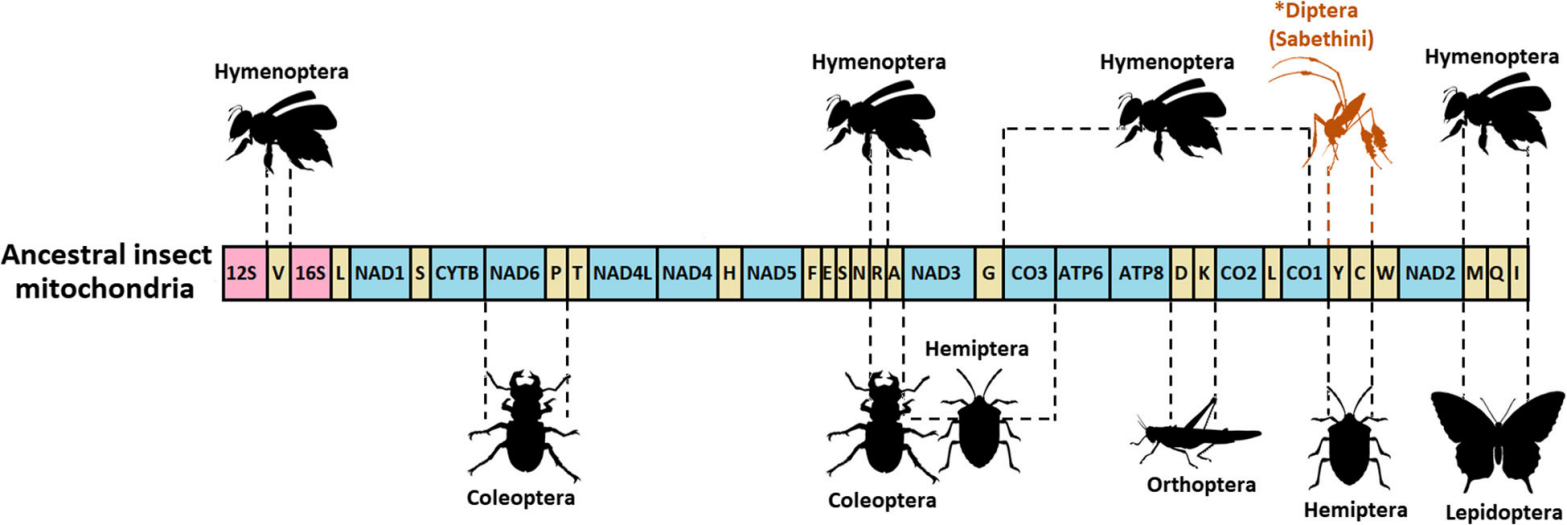
Synapomorphic mitogenome rearrangements found in insect intra-orders mapped along the mitochondrial genome. The translocation of the Y and C tRNA genes, found in the tribe Sabethini, is highlighted in orange. The AT control region was omitted from the representation. Data regarding other insects were obtained from Cameron [1]. This graph is a simplification and do not represent all insect orders that exhibit mitochondrial rearrangements. (Images of insects available on freepik.com. Sabethini image was adapted from a photo of S. Drechsel – diptera.info)

Regarding the translocation found in Sabethini, we searched the literature to determine whether such translocation events are common in other insect orders and mapped them onto mtDNA following Cameron [1] (Fig. 4). We represent only a simplification of few insect orders to exemplify rearrangements occurrence; Embioptera, Mantodea, and Dermaptera also present this phenomenon. There are many groups in which a given rearrangement has been observed in only a single species, but the taxonomic range of these rearrangements is currently unknown. However, it is apparent that mitogenome rearrangements within insects, with some exceptions such as hemipteroids (Phthiraptera, Psocoptera and Thysanoptera) and hymenopterans, are uncommon events.

## Discussion

The tribe Sabethini has remarkable morphological and ecological diversity, unparalleled by other Culicidae tribes. Although widely recognized as a distinct group, the phylogenetic relationships within Sabethini are poorly known [14]. Here, we report for the first time the mitogenomes of five Sabethini species. Knowledge of the mitogenomes of these species can be useful in elucidating phylogenetic relationships within Sabethini. Mitogenomes for the newly sequenced species are similar to those of other *Sabethes* species studied by Aragão et al. [13]. The control region shows greater variability in length than other regions of the mitogenome, as has been observed in other Culicidae species [11]. Similar to other Culicidae [9, 24], the third codon positions has higher AT content than the first and second codons. The transcription hypothesis of codon usage proposed by Sun et al. [25] says that “the high availability of ATPs, along with the lack of other NTPs, leads to the maximization of the use of adenines in the third codon position, increasing the efficiency of the transcription”. Furthermore, purifying selection against deleterious mutations is less severe on the third codon position [11]. Thus, the higher AT content in the third codon position is likely associated with biased usage within synonymous codons.

Regarding gene order, the translocation reported here is likely an autapomorphy of the Neotropical sabethines. This evolutionary event occurred in the Sabethini ancestor at least 75 million years ago based on the dates inferred by Reidenbach et al. [23]. Translocation events within tRNA gene regions have been found in Collembola [26], but very few gene rearrangements have been examined in invertebrates, thus it is difficult to hypothesize about the mechanisms involved in Sabethini mitogenome translocations. Tandem duplications have been proposed to produce translocations [27]. Alternatively, translocations may be produced through the illicit priming of mitochondrial replication by a tRNA molecule (originally proposed by Cantatore et al. [28]). Specifically, after mitochondrial replication is initiated, failure to cleave the tRNA primer from the nascent DNA strand could lead to the ultimate incorporation of a tRNA gene into the mitochondrial genome. It is unclear which model is more likely to have occurred in Sabethini.

Sabethini phylogenetic relationships were robustly resolved by analysis of mitogenomes (Fig. 3). The lineage composed of *Trichoprosopon pallidiventer* and *Runchomyia reversa* was sister to the group of *Sabethes*, *Wyeomyia*, and *Limatus*, which formed a highly supported clade. The phylogenetic tree recovered here supports those found by Reidenbach et al. [23] using six nuclear genes and 80 morphological characters. Our results are also in agreement with those of Aragão et al. [13], where the species of *Sabethes* were monophyletic.

Other contemporary studies of our team lead one to believe in the monophyly of Sabethini based on mitogenomes (Lorenz et al., MS submitted) and pointed to putative autapomorphic wing shape characters (Lorenz & Suesdek, MS submitted). In addition, our study also addressed the phylogenetic placement of the genus *Runchomyia* using DNA sequence data for the first time. This genus is the most difficult to characterize of the sabethines studied here because no clear autapomorphies are known for this genus from any life stage [14]. Along with *Runchomyia*, the genus *Trichoprosopon* represent the most divergent branch within Sabethini, as proposed by Edwards [29]. This group has both morphological synapomorphies with Neotropical taxa and retains many of plesiomorphic characteristics of Oriental and Australian groups [14]. Regarding Sabethini phylogenetic placement within Culicidae, our findings suggest that this tribe is closer to both Culicini and Aedini than to the remaining groups studied. This finding differ from those obtained by Harbach and Kitching [21] and Reidenbach et al. [23] using distinct data sets.

All species studied here are Neotropical, and previous studies strongly support these taxa as a monophyletic group [14, 30]. Species of the genera *Trichoprosopon* and *Sabethes* have been found to be competent to transmit arboviruses to humans and other primates [31]. The genus *Wyeomyia* has also been implicated in the transmission of yellow fever virus [32]. The geographical range of *Sabethes, Trichoprosopon* and *Wyeomyia* is restricted to the Neotropics. Furthermore, Sabethini from the Oriental/Australian region are not vectors of arboviruses. This suggests that vector competence in Neotropical Sabethini for arboviruses may have arisen after Gondwanan separation of the Americas and Africa [14]. The present study also indicates that vector competence to arboviruses may have arisen more than once in Culicidae, in agreement with the hypothesis proposed by Judd [14]. An alternative and parsimonious explanation is that all culicids share vectorial competence but it is expressed unequally across species.

Currently, with representative mitogenomes sequenced from each insect order, no gene rearrangements have been found to be shared between orders [1]. However, we found that synapomorphic mtDNA rearrangements can distinguish groups at taxonomic scales below the ordinal level. Rearrangements occur throughout the mitogenome, with inversions or local rearrangements being most common, whereas translocations are rare. Most gene translocations found in insect mitogenomes are useful for high-level phylogenetic reconstructions [5, 33]. Nevertheless, the results found in the present study suggest that gene order can be informative at lower taxonomic ranks than previously hypothesized, and reinforce the findings from sequence based phylogenetic analysis. The remaining genes in sabethine mitogenomes have the same order as the ancestral hexapodan gene arrangement [34]. Further studies addressing the mitogenomes of Sabethini will provide new insights and additional tools to investigate relationships within a group of mosquitoes of public health importance. In addition, the translocation of two tRNA genes is a remarkable phenomenon that merits investigation with broader taxon sampling.

## Conclusions

In this study, we report the complete mitochondrial genomes of five Sabethini species and the occurrence of a tRNA translocation. The length of these mitogenomes was similar to that of other *Sabethes* species, and the AT-rich control region was the most length variable region. PCG third codon positions had a higher AT content than the first or the second codons, similar to other Culicidae species or animals in general. Regarding gene order, the translocation reported here is unprecedented among Culicidae and represents an autapomorphy for the Neotropical sabethines. Our analyses suggest that gene order can be informative at low taxonomic levels, such as tribes, reinforcing the results from sequence based phylogenetic analysis. The mitogenomes also corroborated the monophyly of Sabethini and provided robust phylogenetic support for the lineage of *Trichoprosopon* plus *Runchomyia* as sisters to a clade composed of *Sabethes*, *Wyeomyia*, and *Limatus*. We suggest that new insights on the phylogeny of the group could be addressed further by using larger taxon sampling, including Oriental and Australian taxa.

## Methods

### Sample collection, data used, and DNA extraction

Mosquito species used in this study share similar habitats, and were captured in the same region of Atlantic forest, São Paulo state, Brazil (24°53′S, 47°51′W), except for *T. pallidiventer*, which was captured in the Amazon forest, Amazonas, Brazil (3°05′S, 60°00′W). Field-collected specimens were identified to species level using morphological keys of Forattini [35] and Lane [36]. Adult females were collected and stored at − 80 °C until DNA extraction. Genomic DNA was extracted from each mosquito individually using a Qiagen DNeasy® Blood and Tissue Kit (Qiagen Ltd., Crawley, UK) following the same procedure as Foster et al. [37]. DNA was examined after electrophoresis on 1% agarose gels and used for PCR amplification. A list of all species used in this study is in Table 2.

**Table 2:** Species used in this study and their mitogenome GenBank accession numbers

Species	References	GenBank
*Sabethes undosus*	This study	MK575488
*Trichoprosopon pallidiventer*	This study	MK575490
*Runchomyia reversa*	This study	MK575487
*Limatus flavisetosus*	This study	MK575482
*Wyeomyia confusa*	This study	MK575492
*Sabethes chloropterus*	[13]	MF957172
*Sabethes glaucodaemon*	[13]	MF957173
*Sabethes belisarioi*	[13]	MF957171
*Aedes aegypti*	[38]	MF194022
*Aedes albopictus*	[39]	KR068634
*Culex quinquefasciatus*	[12]	GU188856
*Culex pipiens*	[40]	KT851543
*Ochlerotatus vigilax*	[41]	KP721463
*Haemagogus janthinomys*	[42]	KT372555
*Nyssorhynchus darlingi* ^a^	[43]	GQ918272
*Kerteszia cruzii* ^a^	[10]	KU551289

^a^ According to Foster et al. [44]

### PCR amplification and sequencing

The mitochondrial genome of each individual was amplified using the one-step long-range polymerase chain reaction method [45]. Reactions were prepared as follows: 25 μL of GoTaq Long PCR Master Mix 2x (Promega, WI), 0.3 μM of each primer (forward and reverse), 1–2 μL of extracted DNA, and sterile water to make up final volume of 50 μL. The primers used for mitochondrial genome amplification follow Hwang et al. [45]: HPK16Saa for the forward strand and HPK16Sbb for the reverse strand. The size of the amplified fragment was approximately 15,300 bp. Thermocycler conditions consisted of: initial denaturation at 94 °C for 2 min, followed by 39 cycles of 94 °C for 15 s, annealing at 65 °C for 20 s, and 65 °C for 15 min, and one final extension cycle at 72 °C for 10 min. The only step that varied between species was annealing temperature: 65 °C for *Wyeomyia confusa* and *Trichoprosopon pallidiventer*, 62 °C for *Limatus flavisetosus*, and 55 °C for *Sabethes undosus* and *Runchomyia reversa*. Amplicons were purified using a DNA Clean & Concentrator kit (Zymo Research, CA) and quantified using a Qubit 2.0 fluorometer (Life Technologies, OR).

### Mitogenome sequence assembly and analysis

Next-generation sequencing was employed to obtain mitochondrial DNA sequences of five Sabethini species. Barcoded libraries were constructed from the PCR products using the Nextera XT DNA Sample Preparation Kit (Illumina, IL) and sequenced on the Illumina MiSeq platform with paired-end 250 bp read chemistry. Raw nucleotide sequences were checked using FastQC software [46]. Mitochondrial genomes were assembled using Mira v4 [47] and Newbler v2.9 software, and visualized in Tablet [48]. Mitochondrial genes were annotated using MITOS [49]. DNA and amino acid (AA) sequence alignments were performed with MEGA 6.06 software [50] and the ClustalW algorithm. Phylogenetic inferences were made using the 13 mitochondrial PCGs from the five Sabethini species sequenced here and other Culicidae available on GenBank (see Table 2). To perform a more comprehensive phylogenetic analysis of the tribe Sabethini, in addition to the newly sequenced samples from our study, we also included in our analysis three species of *Sabethes* previously sequenced by Aragão et al. [13]: *Sabethes chloropterus* (MF957172)*, Sabethes glaucodaemon* (MF957173), and *Sabethes belisarioi* (MF957171). We tested all of the following dataset combinations: amino acids, all DNA sites, or 1st and 2nd codon positions alone. All tests produced the same topology, differing only nodal support. Sequences were concatenated using FASconCAT-G v. 1.04 [51]. All phylogenetic analyses were partitioned based on gene, with gamma-distributed site heterogeneity, and substitution models estimated independently for each partition. Phylogenetic analysis was performed using Maximum Likelihood (ML) with bootstraping = 1000 in RAxML v8.2.11 software [52]. Bayesian phylogenetic inference (BI) was conducted using MrBayes v. 3.2.6 [53] with four runs of eight chains each, run for at least 5 million generations. The tree generated was visualized and customized in FigTree v1.4.3 software [54].

## Data Availability

All the nucleotide data generated in this study were submitted to the NCBI sequence nucleotide database under the accession numbers: MK575482, MK575487, MK575488, MK575490, and MK575492.
